# Rapid Biophysical Characterization and NMR Spectroscopy Structural Analysis of Small Proteins from Bacteria and Archaea

**DOI:** 10.1002/cbic.201900677

**Published:** 2020-01-21

**Authors:** Nina Kubatova, Dennis J. Pyper, Hendrik R. A. Jonker, Krishna Saxena, Laura Remmel, Christian Richter, Sabine Brantl, Elena Evguenieva‐Hackenberg, Wolfgang R. Hess, Gabriele Klug, Anita Marchfelder, Jörg Soppa, Wolfgang Streit, Maxim Mayzel, Vladislav Y. Orekhov, Monika Fuxreiter, Ruth A. Schmitz, Harald Schwalbe

**Affiliations:** ^1^ Institute for Organic Chemistry and Chemical Biology Center for Biomolecular Magnetic Resonance (BMRZ) Johann Wolfgang Goethe University Max-von-Laue-Strasse 7 60438 Frankfurt/Main Germany; ^2^ AG Bakteriengenetik Matthias-Schleiden-Institut Philosophenweg 12 07743 Jena Germany; ^3^ Institute for Microbiology and Molecular Biology Justus Liebig University Giessen Heinrich-Buff-Ring 26 35392 Giessen Germany; ^4^ Faculty of Biology, Genetics and Experimental Bioinformatics Albert Ludwigs University Freiburg Schänzlestrasse 1 79104 Freiburg Germany; ^5^ Biologie II Ulm University Albert-Einstein-Allee 11 89069 Ulm Germany; ^6^ Institute for Molecular Biosciences Johann Wolfgang Goethe University Max-von-Laue-Strasse 9 60438 Frankfurt am Main Germany; ^7^ Department of Microbiology and Biotechnology University of Hamburg Ohnhorststrasse 18 22609 Hamburg Germany; ^8^ Swedish NMR Centre University of Gothenburg P. O. Box 465 40530 Gothenburg Sweden; ^9^ MTA-DE Laboratory of Protein Dynamics Department of Biochemistry and Molecular Biology University of Debrecen Nagyerdei krt 98 4032 Debrecen Hungary; ^10^ Institute for General Microbiology Christian Albrechts University Kiel Am Botanischen Garten 1–9 24118 Kiel Germany; ^11^ Department of Chemistry and Molecular Biology University of Gothenburg Kemigården 4 41296 Gothenburg Sweden

**Keywords:** NMR spectroscopy, proteomics, small proteins, structural biology, structure–activity relationships

## Abstract

Proteins encoded by small open reading frames (sORFs) have a widespread occurrence in diverse microorganisms and can be of high functional importance. However, due to annotation biases and their technically challenging direct detection, these small proteins have been overlooked for a long time and were only recently rediscovered. The currently rapidly growing number of such proteins requires efficient methods to investigate their structure–function relationship. Herein, a method is presented for fast determination of the conformational properties of small proteins. Their small size makes them perfectly amenable for solution‐state NMR spectroscopy. NMR spectroscopy can provide detailed information about their conformational states (folded, partially folded, and unstructured). In the context of the priority program on small proteins funded by the German research foundation (SPP2002), 27 small proteins from 9 different bacterial and archaeal organisms have been investigated. It is found that most of these small proteins are unstructured or partially folded. Bioinformatics tools predict that some of these unstructured proteins can potentially fold upon complex formation. A protocol for fast NMR spectroscopy structure elucidation is described for the small proteins that adopt a persistently folded structure by implementation of new NMR technologies, including automated resonance assignment and nonuniform sampling in combination with targeted acquisition.

## Graphical abstract

**Reading expands horizons**: Structural analysis of small proteins encoded by previously overlooked small open reading frames (sORFs) is achieved by using NMR spectroscopy. A protocol is established for fast NMR spectroscopy structure screening, including optimized sample preparation for systems of this size (14–78 aa), and determination of the structural conformations adopted by the systems.

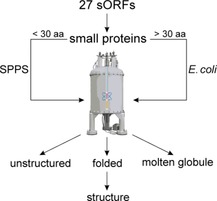

## Introduction

For a long time, technical limitations in detection and assumptions in the automated gene annotation tools that were too strict prevented the identification of peptides and small proteins encoded by small open reading frames (sORF). Only after several small proteins were identified in different organisms and shown to be encoded by sORFs was the previous assumption that an ORF should have a minimum length of 100 codons called into question. In the last decade, increasing efforts to identify and study peptides and small proteins and their functions with diverse computational and biochemical methods, including ribosome profiling and mass spectrometry optimized for peptides,^1^, ^2^ have resulted in paradigm‐shifting discoveries. An increasing number of small proteins have been found to play important roles in a broad range of cellular functions, including cell division, morphogenesis, and stress response.^3^

The nomenclature and definition of the maximal size of the small proteins encoded by sORFs varies between 50 and 100 amino acids.^4^ Typically, peptides are distinguished from proteins by the shorter length of their chain. The exact cutoff is vague, but often set to 50–60 amino acid residues.^3^ In addition, many names are used for the small‐sized proteins, including micropeptide, microprotein (μ‐protein), miniprotein, and small protein. Even the same name is sometimes defined differently, such as the term microprotein.^5^, ^6^, ^7^ To overcome this diversity, we herein refer to Storz et al.,^3^ who apply the term “small proteins”, and set the upper sequence length to be fewer than 80 residues.

Small proteins were found in all three domains of life (Archaea, Bacteria, and Eukarya). Small proteins can be categorized according to their mode of biosynthesis as either geneencoded/ribosomally synthesized (RPs) or non‐gene‐encoded/nonribosomally synthesized peptides (NRPs). In contrast to NRPs, RPs are restricted to 22 (including selenocysteine and pyrrolysine) proteinogenic amino acids and often undergo diverse post‐translational modifications (PTMs), such as hydroxylation, methylation, halogenation, prenylation, acylation, thioether and thioester crosslinking, epimerization, and macrocyclization.^8^, ^9^, ^10^ These modifications potentially decrease their structural flexibility and favor structure formation, increase their stability by preventing degradation by proteases, and provide additional regulatory functions. Ribosomally synthesized and post‐translationally modified peptides (RiPPs) often bind to a structured interaction partner. Prominent examples include the antibiotics thiostrepton and micrococcin from the thiopeptides family that bind to the GTPase‐associated region (GAR) of the 50S ribosome and inhibit translation.^11^ Peptides with antibacterial properties, also known as antimicrobial peptides (AMPs), are essential as therapeutic antibiotics for drug development.^12^, ^13^, ^14^, ^15^

Many small proteins, if overexpressed, are toxic for bacteria.^16^, ^17^, ^18^ In *Escherichia coli*, they are often hydrophobic and contain single transmembrane helices. This promotes inner membrane insertion and frequently the loss of membrane potential.^16^ Among pathogenic bacteria, important examples include the phenol‐soluble modulins (short, amphipathic, α‐helical peptides in staphylococci).^19^ Many of the small toxic proteins belong to the class I toxin/antitoxin loci, in which expression of a generally stable small and hydrophobic toxin (<60 aa) is inhibited at the level of translation by a *cis*‐encoded antisense RNA.^18^, ^20^, ^21^, ^22^ Apart from well‐described examples, for example, essential functions in persister cell formation,^23^, ^24^ the functional roles of predicted chromosomally encoded toxin/antitoxin loci often remain unknown. They might be involved in genomic integrity, slowdown of growth under stress conditions or simply act as “selfish DNA”.^18^

The photosynthetic apparatus contains a number of small proteins with functions that are conserved from cyanobacteria to higher plants; some with fewer than 50 amino acids play a role in photosynthetic electron transport (Cyt*b*_6_ *f* complex; *petL*, *petN*, *petM*, *petG* 25, 26, 27), in photosystem II (genes *psbM*, *psbT* (*ycf8*)*, psbI*, *psbL*, *psbJ*, *psbY*, *psbX*, *psb30* (*ycf12*), *psbN*, *psbF*, *psbK* 28, 29), in photosystem I (*psaM, psaJ, psaI* 30), or with accessory functions in photosynthesis (*hliC)*.^31^ With 29 amino acids, the cytochrome *b*_6_ *f* complex subunit VIII, encoded by *petN*, is the shortest of these proteins.^27^

However, elucidation of the function of small proteins remains a challenging task and the structural characterization of small proteins can help in elucidating their molecular mechanisms of action. Despite their small size, which has impeded their recognition, small proteins have been found in every living cell.^3^ Their regulatory function encompasses a wide range, including regulation of the enzyme activity of both membrane‐bound and cytosolic proteins. They can further induce or modulate the conformation of larger proteins or other biomolecules, and thus, can be part of complex signal transduction processes.

**Figure 1:**
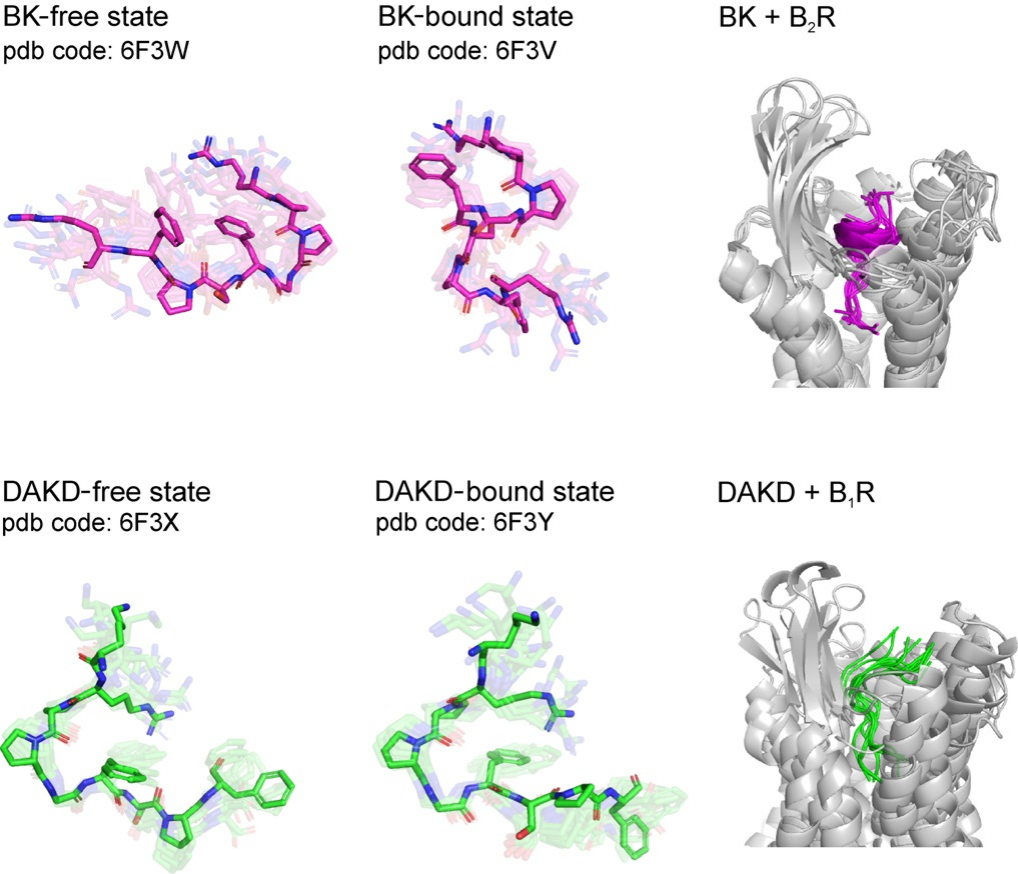
A comparison of the structures of DAKD and BK in free and BR‐bound forms determined by means of NMR spectroscopy.^12^

Small proteins with cellular function are not exclusively found in bacteria or archaea, but several eukaryotic small proteins have been identified and their functions studied. One example is the selective interaction of the neuropeptide bradykinin (BK), a peptide agonist (nine amino acids), with the human BK G protein coupled receptor, a drug target for cardiovascular regulation. Joedicke et al. investigated the conformational differences of the analogous peptides desArg10‐kallidin (DAKD) and BK induced by interaction with the target, to understand their specific binding behavior.^12^ Even though the peptides are very similar, their behavior is substantially different. The two small proteins show differences upon receptor binding: the free and bound forms of the kallidin peptide are very similar, whereas the conformation of BK is rearranged upon binding to its receptor. The kallidin peptide binds in an open conformation, whereas parts of the BK peptide chain adopt specific folded conformations. These results show that receptor specificity of peptide ligands is dependent on the conformational and chemical space of peptides. Conformational changes are not necessarily required to achieve specific peptide–receptor interactions (Figure 1).

Small proteins are most often unstructured. In the case of residual structure, the preferred structural motifs are often restricted to α‐helical elements. Further short motifs that mediate specific protein interactions can be identified from the sequence, but they might require longer flanking regions to function.^32^ The function of many small proteins is often related to their ability to undergo a disorder‐to‐order transition; thus adopting a folded state upon interaction with their biological targets. However, function might not require such a disorder‐to‐order transition. The intrinsically disordered proteins (IDPs), for example, lack a persistent three‐dimensional folded structure and can still be functional, in particular, in transcriptional and translational regulation and in signal transduction within the cell.^33^, ^34^

The number of identified small proteins is currently rapidly growing due to novel approaches, including high‐resolution ribosome profiling with stalled initiation complexes.^35^, ^36^ Hence, more efficient methods are required to investigate the structure–function relationships of small proteins.

Elucidation of the relationship between the protein sequence, structure, and function is a key part of computational biology studies. Typical prediction methods are, however, limited to inherently structured proteins and are not convenient for disordered proteins, the fold and stability of which potentially depend on their biological target. The estimation of a favorable, stable, bound conformation gives an important insight into the probability of the protein folding upon complex formation; this might be related to its function.^37^ These prediction methods,^38^, ^39^, ^40^ which were initially developed for IDPs, were successfully applied to the set of 27 small proteins characterized in this study. Computational prediction results were accompanied by experimental solution‐state NMR spectroscopy data of small proteins in their free form.

NMR spectroscopy is an experimental technique that allows the determination of structure and dynamics of isolated small proteins in solution, as well as within complexes with diverse cellular components.^41^, ^42^ NMR spectroscopy structure determination has been successfully integrated into other structural genomic approaches to increase the number of resolved 3D proteins structures.^43^, ^44^ The quality assessment of the determined NMR protein structure can be further performed by the Worldwide Protein Data Bank (wwPDB) validation report.^45^, ^46^

Furthermore, NMR spectroscopy provides an unbiased readout of the folding state of a protein. The method can equally well describe the structure of folded proteins and of partially folded and unstructured (random coil) states.^47^ This potential of unbiased characterization is particularly important for the structural investigation of small proteins because many are unlikely to adopt a stable folded conformation if isolated in solution. Furthermore, because the proteins of interest (POI) are small, their NMR spectroscopic investigation is, in principle, rather straightforward, including rapid de novo structure determination.

To perform a routine secondary‐structure screening of a high number of small proteins, the sample preparation strategy is required to be fast and precise. Hence, we describe the optimization of a pipeline to conduct structural studies of more than 20 different small proteins, which we investigate within a priority program on small proteins (SPP2002) that has been funded by the German Research Foundation (DFG) since 2018. We have established a routine work protocol and screened 27 small proteins for secondary structure. Within this screening, we observed all possible conformations: 1) entirely unstructured proteins (random‐coil state): 2) proteins with a fluctuating amount of residual structure, but no defined tertiary fold (molten globule), as well as specific structures with only lowly populated excited states; and 3) structured proteins.^48^, ^49^ We report herein on the protocol for structure determination of one exemplary small protein (SP‐22; PDB ID: 6Q2Z; BMRB ID: 34334).^50^ For high‐throughput structural approaches, a reduction of measurement time is advantageous. We used the nonuniform sampling (NUS) approach in combination with targeted acquisition (TA) and a new multidimensional decomposition (MDD) signal processing technique.^51^, ^52^, ^53^

## Results and Discussion

### Expression and purification strategy

**Figure 2:**
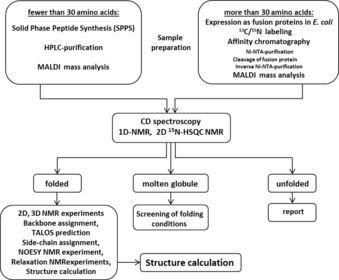
Workflow for NMR spectroscopy structural investigations of small proteins, including synthesis/expression and purification steps. CD: circular dichroism.

We established an efficient workflow for the characterization of conformation and dynamics of small proteins (Figure 2). Within this workflow, small proteins containing fewer than 30 amino acids were synthesized by means of SPPS and purified by reversed‐phase HPLC. Small proteins containing more than 30 amino acids were heterologously expressed in *E. coli*. For NMR spectroscopy and structure determination, ^15^N‐ and ^15^N/^13^C‐labeling schemes were performed by using M9 minimal medium containing ^15^N‐labeled ammonium chloride and ^13^C‐labeled glucose. The small POI were expressed as N‐terminal small ubiquitin‐related modifier (SUMO) fusion proteins, which enhanced expression, improved solubility, masked possible toxicity, and reduced the proteolytic degradation of recombinant proteins. In particular, the SUMO tag allowed the generation of the exact desired N terminus of POI.^54^ The SUMO protease cleaved the tertiary SUMO folding motif without additional amino acids.^55^ This remarkable ability was very important for small POI, in which every additional amino acid can influence the structural properties. Addition of a hexahistidine tag to the SUMO fusion facilitated purification by means of tandem Ni‐NTA affinity chromatography^56^ followed by size exclusion chromatography. In case a construct did not express with the SUMO tag (e.g., for small protein SP‐24), a hexahistidine tag was linked to the protein sequence through a thrombin cleavage recognition site. Subsequent purification included Ni‐NTA affinity chromatography followed by size exclusion chromatography to separate the small protein from the cleaved hexahistidine tag. For POI that incorporated metal ions (e.g., Zn‐binding proteins), the Ni‐NTA affinity chromatography purification strategy was changed to affinity chromatography with glutathione‐sepharose beads^57^ by adding an N‐terminal glutathione S‐transferase (GST) fusion tag. Removal of the GST fusion partner after tobacco etch virus (TEV) cleavage was easily achieved by using size exclusion chromatography. The additional amino acids remaining on the N terminus after cleavage are glycine and alanine, with potential small influence on the structure in the elongated small protein. Identification and purity of the produced small POI were confirmed by means of SDS‐PAGE analysis and mass spectrometry (MALDI).

### Rapid secondary‐structure determination

**Figure 3:**
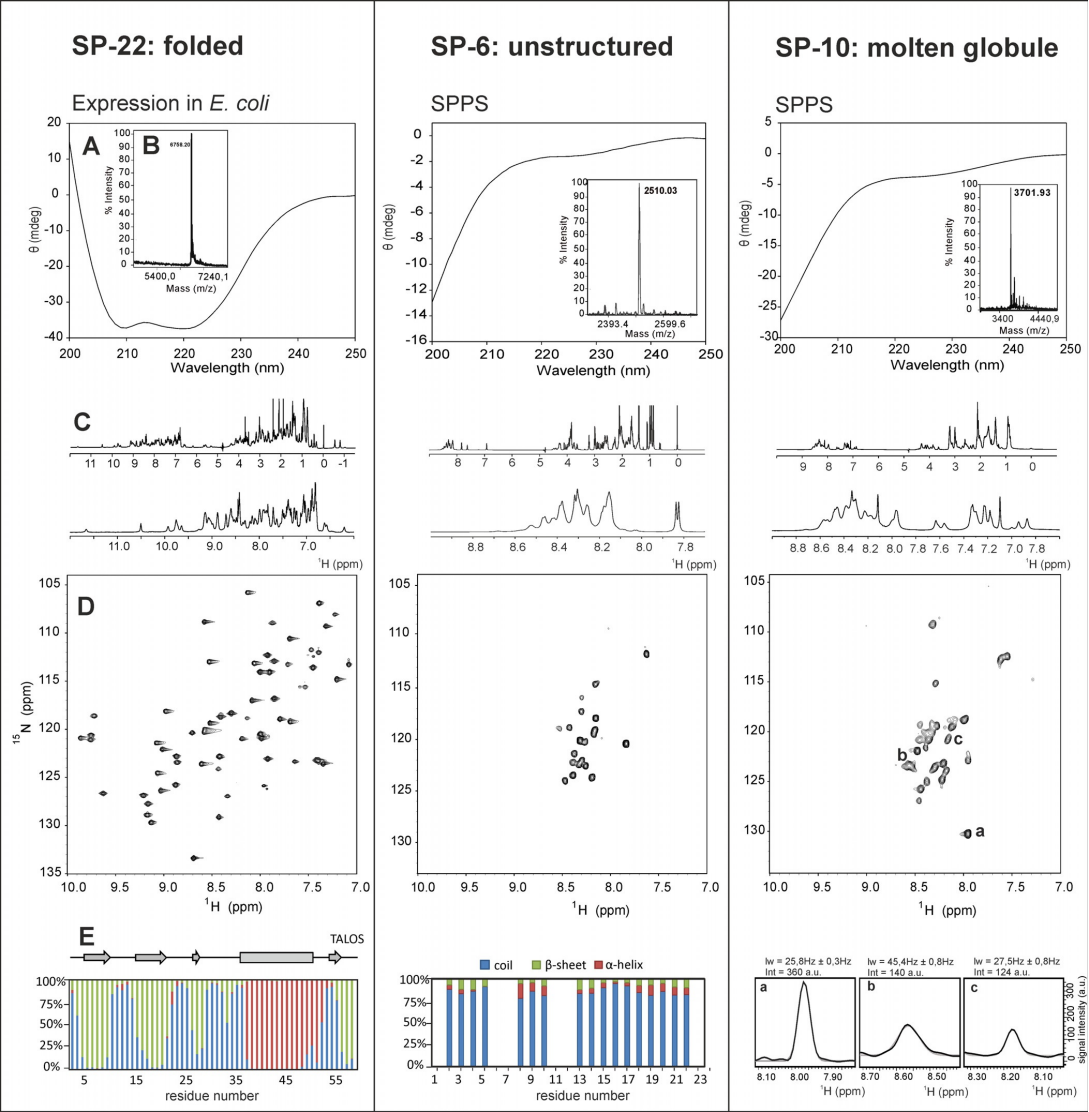
Left: SP‐22 small protein from *Haloferax volcanii*; middle: SP‐6 small protein from *Methanosarcina mazei*; right: SP‐10 small protein from *Sinorhizobium fredii*. A) CD spectra in phosphate buffer at pH 7. B) MALDI mass analysis of the purified small protein. C) The 1D ^1^H NMR spectra with an enlargement of the amide proton region. D) The 2D ^1^H,^15^N HSQC spectra at 600 MHz, 298 K. E) Left and middle: TALOS secondary‐structure prediction of the residues that are classified as “good”; right: expanded regions from the 2D ^1^H,^15^N HSQC spectrum for three signals showing differences in line width and intensity of the NMR signals, which are characteristic for molten globule‐type conformational behavior. These results agree perfectly with calculations by the Espritz method in free‐form propensity of structures SP‐22 (73.3 % folded), SP‐6 (0 % structured), and SP‐10 (61.3 % molten globule).

After protein purification to a purity above 95 %, the POI were analyzed by means of CD spectroscopy (Figure 3 A) and 1D ^1^H NMR spectroscopy (Figure 3 C) to obtain global information about their secondary structures. Two‐dimensional ^1^H,^15^N HSQC NMR spectra, which can nowadays often be recorded by using the low natural abundance of 0.3 % in ^15^N, allow direct structural readout. The dispersion of the detected backbone amide signals is a structural fingerprint of the protein. Their position, intensities, and line widths are markers for the conformational state of the investigated protein: structured (Figure 3 D, left), unstructured (Figure 3 D, middle), or partially structured and undergoing millisecond intermediate exchange (Figure 3 D, right). Chemical shift analysis by TALOS allows a detailed and reliable site‐specific secondary‐structure prediction (Figure 3 E).^58^ TALOS analysis requires the full backbone resonance assignment of the small protein, for which further 3D NMR spectroscopy experiments on a ^15^N,^13^C‐labeled sample are needed.

The throughput for structural screening of small proteins is limited by the NMR spectroscopy measurement time needed to record multidimensional spectra, their resonance assignment, and subsequent structure determination. Depending on the protein concentration, the data needed for determining a protein structure require at least three weeks of measurement time followed by data analysis. The reduced signal overlap achieved by the highly resolved multidimensional spectra is crucial for (manual or automated) signal assignment and structure calculation, but this procedure is very time‐consuming, and thus, not applicable for the screening of a large set of small proteins (and likewise for unstable proteins). The NUS approach provides a solution because it strongly reduces the experimental time and/or allows for higher resolution without the loss of important information, if the obtainable signal‐to‐noise ratio is sufficient.^59^

In addition, a TA and a new MDD signal‐processing technique^51^, ^52^, ^53^ applied for nonuniform sampled data dramatically reduces the NMR spectroscopy measurement time and simplifies the evaluation of the spectra. In combination with automated methods for signal list generation and resonance assignment (such as FLYA),^60^ this method is suitable for the rapid structural screening of small proteins.

Structure elucidation (by using CYANA with fully automated NOESY crosspeak assignment)^60^, ^61^, ^62^, ^63^ can be performed after completing backbone and side‐chain NMR resonance assignment. The final structure calculation generally includes NOE data, hydrogen bonds, ample dihedral angle restraints based on TALOS predictions, and ^3^ *J*(H^N^,H^α^) coupling constants.^64^

### Application of the pipeline for 11 bacterial small proteins and 17 archaeal small proteins

In total, 27 different small proteins, ranging from 14 to 78 amino acids, were screened. Ten small proteins were synthesized by means of SPPS (14–31 amino acids) and 17 small proteins were expressed in *E. coli* (38–71 amino acids). Of these, three small proteins could not be expressed, for four we initially obtained degraded samples, and for one further small protein structural investigation was not possible because of insolubility due to its hydrophobicity. We rapidly assessed the secondary‐structure screening of the remaining 19 small proteins by means of CD and NMR spectroscopy.

**Figure 4:**
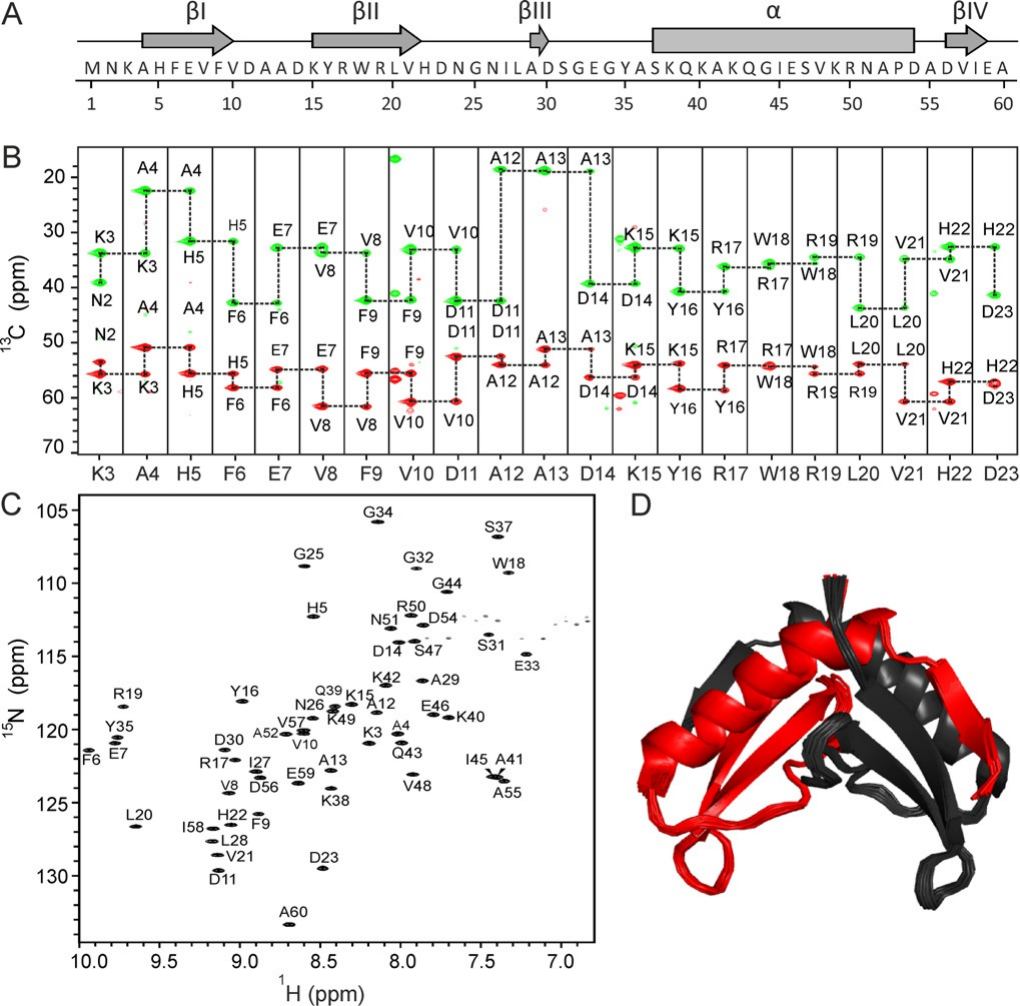
Structural characterization of SP‐22 small protein from *H. volcanii*. (PDB ID: 6Q2Z; BMRB ID: 34334). A) The amino acid sequence and the schematic representation of the secondary‐structure elements based on the solution‐state NMR spectroscopy structure. B) Sequential assignment for residues K3 to D23. The 3D HNCACB NMR spectrum was recorded at 700 MHz, 298 K; it contains 5 mm protein, 50 mm sodium phosphate buffer pH 7.5, 100 mm NaCl, 5 % D_2_O, 0.5 mm DSS. C^A^ are shown in red and C^B^ are highlighted in green. C) ^1^H,^15^N Best TROSY spectrum (600 MHz) of 5 mm small protein in 50 mm sodium phosphate buffer, pH 7.5, 100 mm NaCl, 95 % H_2_O/5 % D_2_O at 298 K. Backbone resonance assignment is indicated. D) Solution‐state NMR spectroscopy structure of SP‐22 protein. Ribbon representation of the best 20 structures is shown as a symmetrical dimer. The monomer consists of one α helix and four β‐sheet regions. Black and red represent two monomeric subunits. The figure was generated with PyMOL.^50^

All small proteins with fewer than 30 amino acids were found to be unstructured. Six small proteins adopted a molten globule conformation. Thus, almost all small proteins with fewer than 30 amino acids were shown to be predominantly unstructured, whereas those between 30 and 80 amino acids tended to adopt a structured or partially structured conformation. The small size of the proteins clearly seems to restrict the possible structural motifs. Nevertheless, structures might be adopted for these small proteins upon interaction with their biological targets. Furthermore, five small proteins (all above 50 amino acids) were found to be structured or to contain a partially structured conformation. Interestingly, two of the structured small proteins contained at least one Zn^2+^‐binding motif, which might lead to structural stability of these proteins. The structural investigation of these small proteins was conducted by using ^13^C/^15^N isotope labeling (Table 1 and Table S1 A–D in the Supporting Information). Structure determination of one folded small protein has been published,^50^ and the requirements for structure determination and the quality of structure are exemplarily shown for the SP‐22 small protein from *H. volcanii* (Figure 4).

**Table 1:** Structural analysis validated by means of NMR and CD spectroscopy of the small proteins screened in this study. The table is shown with an ascending molecular weight of small proteins. A full overview can be found in Table S1 A–D.

ID	aa	MW	Microorganism	CD and NMR
		[kDa]		structural analysis
SP‐1	14	1.6	*Bradyrhizobium japonicum*	unstructured
SP‐2	14	1.8	*Sinorhizobium meliloti*	unstructured
SP‐3	18	1.9	*Dinoroseobacter shibae*	unstructured
SP‐5	23	2.6	*M. mazei*	unstructured
SP‐6	23	2.8	*M. mazei*	unstructured
SP‐7	27	2.9	*S. meliloti*	unstructured
SP‐8	28	3.1	*M. mazei*	unstructured
SP‐9	29	3.1	*M. mazei*	unstructured
SP‐10	31	3.7	*S. fredii*	molten globule
SP‐11	38	4.0	*H. volcanii*	molten globule
SP‐12	39	4.5	*Bacillus subtilis*	molten globule
SP‐13	43	4.8	*S. fredii*	molten globule
SP‐19	51	5.7	*H. volcanii*	structured
SP‐21	59	6.5	*H. volcanii*	structured
SP‐22	60	6.7	*H. volcanii*	structured
SP‐23	61	6.9	*H. volcanii*	molten globule
SP‐24	61	7.1	*M. mazei*	structured
SP‐25	61	7.2	*H. volcanii*	molten globule
SP‐27	78	8.1	*H. volcanii*	partially structured

For eight small proteins (four from archaea and four from bacteria), we could not establish protocols for expression. They range from 18–71 amino acids, and thus, do not show any bias in size. At this point, we restricted ourselves to study systems for which the above described workflow could easily be applied. Further efforts for some of the sequences are currently pursued, but beyond the scope of this contribution.

The small proteins screened in this study belong to different bacterial and archaeal organisms (Table S1 D), but the approach is not limited to prokaryotic systems and can be applied to eukaryotic small proteins, as well in future investigations.

### Bioinformatic sequence‐to‐structure analysis

A bioinformatic sequence‐to‐structure analysis provides a first readout for possible secondary structure of the small protein. For this purpose, we used fast online structure prediction tools, including pep‐FOLD,^65^, ^66^ PEPstrMOD,^67^, ^68^ and SWISS‐MODEL.^69^, ^70^, ^71^, ^72^, ^73^ The sequence‐based secondary‐structure prediction for small proteins containing fewer than 30 residues generally predicted α‐helical regions. These predictions, however, were not confirmed by CD and NMR spectra, which actually reported a completely unstructured state (Table 1). The s2D method,^40^ however, which is based on NMR chemical shifts, predicted 12 out of 20 sequences dominantly (>50 %) coiled or disordered (Table S2). In addition, SWISS‐MODEL secondary‐structure predictions, which can be applied to small proteins with more than 30 amino acids, can provide a good starting point if homologous domains or structures with high sequence identity already exist and can therefore be used for generating a structural model of the small protein (Figure S1). SWISS‐MODEL and the s2D method can be used together to predict the structure and dynamics/disorder of small proteins.^74^

The second layer to link the sequence to structure is to estimate the preference for a folded, stable conformation. A series of experimental and computational results indicate that approximately 40 % of eukaryotic proteomes do not adopt a well‐defined structure, but interconvert between alternative conformations in their native state.^75^ IDP regions or whole domains often serve regulatory roles through interaction partners, which may induce their folding.^33^ These findings have triggered the development of a plethora of algorithms to predict the preference for a folded tertiary structure versus intrinsic disorder. The Espritz NMR spectroscopy method with the highest performance, relative to that of NMR data,^39^ predicted that 11 out of the 19 sequences analyzed in our study contained more than 50 % structured residues and 4 sequences had 30–50 % disordered residues (Tables 2 and S4). This was comparable to the results obtained by a meta approach based on a combination of predictors (PrDOS, DisoPred2, VSL2, IUPred;^38^ Table S3). Except for short sequences with fewer than 25 amino acids, disorder predictions were in accordance with our experimental data (Table 1). This problem was due to the compositional bias in disordered regions,^76^ which was difficult to evaluate in short sequences. Small proteins were found to be mostly unstructured, similarly to the protein sequences that were prone to “default” degradation by the 20S proteasomes,^77^ which significantly decreased the half‐life of these proteins.^78^ We found that the propensity of predicting residues to be in structured regions paralleled the experimentally detected increasing order in molten globule states and structured small proteins (Tables 2 and S4).

**Table 2:** Espritz NMR spectroscopy predictions of sequences in free form. Dynamic transitions induced by interactions were computed by using the FuzPred method with a reference to the Espritz NMR spectroscopy free form. Small proteins were combined in classes with respect to experimental secondary‐structure screening analysis.

	ID	aa	Free form [%]		Bound form [%]	
			Structured	Disordered	Structured	Disordered
folded	SP‐19	51	68.6	31.4	92.2	7.8
	SP‐21	59	71.2	28.8	84.7	15.3
	SP‐22	60	73.3	26.7	100	0
	SP‐24	61	100	0	100	0
	SP‐27	78	100	0	100	0
molten	SP‐10	31	61.3	38.8	93.5	6.5
globule	SP‐11	38	73.7	26.4	100	0
	SP‐12	39	20.5	79.4	41	59
	SP‐13	43	53.5	46.5	67.4	32.6
	SP‐23	61	67.2	32.8	78.7	21.3
	SP‐25	61	19.7	80.4	75.4	24.6
unstructured	SP‐1	14	78.6	21.4	100	0
	SP‐2	14	21.4	78.6	78.6	21.4
	SP‐3	18	0	100	61.1	38.9
	SP‐5	23	21.7	78.2	87	13
	SP‐6	23	0	100	52.2	47.8
	SP‐7	27	0	100	63	37
	SP‐8	28	0	100	57.1	42.9
	SP‐9	29	79.3	20.7	100	0

Disordered peptides may be stabilized by interactions through “coupled folding to binding” mechanisms.^79^ Conformational heterogeneity, however, can also be retained in complexes. Complex formation then does not induce (complete) structure.^37^ Thus, it is important to predict the probability of folding upon binding. For this, we used the FuzPred algorithm.^80^ This method is based on the local compositional and dynamical bias in interacting regions, which can be quantified without prior knowledge of specific interaction partners (protein, RNA, DNA, metabolite, or other possible partners). The method has been validated on more than 2000 protein complexes in the PDB, including both structured and disordered assemblies. Based on FuzPred predictions, the structure propensity increased by interaction partners, that is, disordered regions tended to fold upon binding (Tables 2 and S2 C). The propensity of residues to adopt alternative conformations or exhibit conformational exchange in the bound state was fewer than 20 % in structured sequences and fewer than 35 % in molten globule states. In our screening, SP‐12 was the only sequence that was predicted to dynamically fluctuate in the presence of an interaction partner, possibly owing to the presence of tandem, multivalent motifs. Degradation‐prone small proteins could also gain structure upon binding, which might prolong their lifetimes.^81^ Unstructured sequences often still exhibit a considerable fraction of disordered residues in the bound state. These regions might be involved in further interactions or regulation of larger, supramolecular assemblies.^82^, ^83^

### Screening of folding conditions

One of the main tasks in structural proteomics is to identify the optimal protein folding conditions suitable for structure elucidation by means of NMR spectroscopy. This procedure includes screening of the protein stability and ability to fold at different pH, buffers, salt concentration, and temperature. For a first rapid screening, we used CD spectroscopy because these experiments benefited from low sample costs (no isotope labeling needed) and fast implementation. The CD profile is indicative of unstructured and folded protein and can provide information about the presence of structural elements. NMR spectroscopy, however, can provide more precise and detailed information, and is the only possibility for screening proteins with different conformational states or partially folded molten globule states.

### Influence of pH and salt concentration on protein conformation

The protein SP‐23 from archaeon *H. volcanii*, which is part of our screening, shows conformational changes that are dependent on stress and high salt conditions and is therefore an excellent example to show the influence of varied conditions. Changes in pH can dramatically affect the 2D ^1^H,^15^N HSQC protein spectra. The expected number of signals is observed at neutral pH 7, whereas a decrease to more acidic pH 6 leads to double the amount of signals; this clearly indicates the presence of conformational heterogeneity under these conditions. In contrast, an increase of the pH to more basic pH 8 leads to faster amide proton exchange and results in signal loss in the spectra (Figure S2 A). This effect is observed in secondary‐structure elements that undergo dynamic opening or closing events and, at basic pH, is difficult to correlate to structure or function of the POI.

One representative conformational marker in the 2D ^1^H,^15^N HSQC spectrum is the tryptophan indole side‐chain signal, which appears at an isolated spectral region downfield at around *δ*=10 ppm (^1^H). Although tryptophan occurs only once in the protein sequence, the spectrum shows a doublet signal at all screened pH conditions; thus suggesting the presence of a minor populated conformation.

Because this protein is involved in high salt regulation, we monitored the conformational changes upon increasing the NaCl concentration (Figure S2 B). A series of 2D ^1^H,^15^N HSQC spectra were recorded at pH 7, with NaCl concentrations ranging from 0 to 1 m. The 2D ^1^H,^15^N HSQC spectrum underwent significant changes upon addition of 100 mm NaCl, resulting in the loss of signals and appearance of new signals. A further increase of the salt concentration to 300 mm led to chemical shift perturbations (CSPs) of almost all of the signals, and thus, was indicative of charge screening of the protein. A substantial number of new, less intense signals appeared, which indicated an upcoming low populated conformation. An even further increase in the salt concentration only led to minor CSPs and reached a steady state at 500 mm salt concentration.

### Temperature dependence on folding

In contrast to stable folded proteins with linear temperature‐dependent amide chemical‐shift changes (indicative of the presence or absence of hydrogen bonds), small proteins in molten globule states and in conformational equilibria are most often sensitive to changes in temperature, which may impact their preferred structural motif. The proper conditions for temperature‐induced folding can thus be screened easily and rapidly by following the amide signal changes in a series of 2D ^1^H,^15^N HSQC spectra measured at different temperatures.

For example, for one of the screened small proteins, SP‐21, we could detect temperature‐dependent conformational heterogeneity (Figure S3). Monitoring the changes of the amide chemical shift and its signal intensity upon temperature variation in 2D ^1^H,^15^N HSQC spectra provided information about the structural changes and the equilibrium shift between the conformations adopted at different temperatures.

### NMR spectroscopy time‐saving methods

If the screened small protein appears to be folded, the following steps for elucidating the solution‐state NMR spectroscopy structure are conventionally rather time‐consuming. To speed up these steps, several methods have been developed that shorten the experimental time and processes for resonance assignment and NOESY crosspeak assignment. Reduction of the NMR spectroscopy measurement time can be achieved by applying TA in combination with the MDD signal‐processing technique on NUS data.^51^, ^52^, ^53^ Subsequently, a method for automated resonance assignment, FLYA,^60^ can be performed by using the program CYANA for automated NOESY crosspeak assignment and structure calculations.^60^, ^61^, ^62^, ^63^ The benefit of using this technique is exemplified for the SP‐22 small protein.

The NMR spectroscopy experiments used for manual assignment were also used as input for the automated assignment with FLYA. The manually determined chemical‐shift assignment was used as a gold standard. Most of the automated assignments were in good agreement with the manual assignment (Figure S4 A), and thus, demonstrated the high quality of the automated FLYA assignment method. Furthermore, FLYA, as a part of the TA procedure, could be performed in a few minutes, which was a dramatic reduction in comparison with the manual assignment process, which generally takes roughly two weeks. The set of experiments for the backbone assignment was acquired by using NUS and the TA technique and included the following 3D heteronuclear NMR spectroscopy experiments: HNCO, HN(CO)CA, HNCA, HN(CO)CACB, HNCACB, and HN(CA)CO. Analysis of the quality of the recorded spectra was performed by using FLYA. The manual process for structure determination by means of NMR spectroscopy, based on time‐consuming, conventional NMR spectroscopy methods, was used as a reference. The measurement time was reduced by more than 20 times (5 days for conventional methods and 4.5 h for TA in combination with MDD) without significant loss of spectral quality (Figures S4 and S5).

## Conclusion

NMR spectroscopy can serve as a powerful tool for structural screening of small proteins to provide detailed information about their conformations. We showed that optimization of the folding conditions for structure determination of proteins could profit from rapidly performed CD and NMR spectroscopy experiments.

Using these methods, we demonstrate that most of the investigated small proteins do not adopt a persistent conformation and exist mostly in an unstructured or partially structured state. This is in perfect agreement with the results obtained by the Espritz NMR spectroscopy disorder prediction method performed for small proteins in the free state. Very likely, their small size restricts them to adopt a stable fold. However, these small proteins may need an interaction partner to gain a structured conformation through complex formation. FuzPred predictions of the structure propensity show an increased trend for folded conformation upon intermolecular interaction. Specific interaction partners have to be identified and their induced conformational changes have to be investigated in the future.

The measurement time needed for structural analysis can be reduced by using TA, in combination with the MDD signal processing technique, applied on NUS data. This protocol was successfully applied on two small proteins. The implemented automated FLYA assignment simplifies the evaluation of the spectra and speeds up the structural screening of small proteins.

The investigation of small proteins represents a new field of research that has increased rapidly in recent years. Genomic research will predict and generate a large number of new small proteins. For these small proteins, the function needs to be determined. Akin to the field of IDPs, the structure–function paradigm of molecular biology is challenging. Thus, it is important to holistically investigate these small proteins. Herein, we showed that NMR spectroscopy could be applied to rapidly screen for secondary structure and conformational properties of small proteins, including detailed structural analysis for those proteins that, despite being small, adopted a persistent three‐dimensional fold.

## Conflict of interest

*The authors declare no conflict of interest*.

## Supporting information

As a service to our authors and readers, this journal provides supporting information supplied by the authors. Such materials are peer reviewed and may be re‐organized for online delivery, but are not copy‐edited or typeset. Technical support issues arising from supporting information (other than missing files) should be addressed to the authors.

Supplementary
