# Supplementary material for: Rapid Biophysical Characterization and NMR Spectroscopy Structural Analysis of Small Proteins from Bacteria and Archaea
Source: Chembiochem. 2020 Jan 21;21(8):1178–87. doi: 10.1002/cbic.201900677 (PMC7217052; doi:10.1002/cbic.201900677)
Supplement: Supplementary file 1 — Supplementary [file CBIC-21-1178-s001.pdf]

CHEMBIOCHEM

Supporting Information

Rapid Biophysical Characterization and NMR Spectroscopy Structural Analysis of Small Proteins from Bacteria and Archaea

Nina Kubatova,[a] Dennis J. Pyper,[a] Hendrik R. A. Jonker,[a] Krishna Saxena,[a] Laura Remmel,[a] Christian Richter,[a] Sabine Brantl,[b] Elena Evguenieva-Hackenberg,[c] Wolfgang R. Hess,[d] Gabriele Klug,[c] Anita Marchfelder,[e] Jörg Soppa,[f] Wolfgang Streit,[g] Maxim Mayzel,[h] Vladislav Y. Orekhov,[h, k] Monika Fuxreiter,[i] Ruth A. Schmitz,*[j] and Harald Schwalbe*[a]

cbic_201900677_sm_miscellaneous_information.pdf

Supporting Information

Table of contents

Table S1 A. Overview of the amino acid sequences of small proteins screened in this study..................... 2

Table S1 B. Overview of the origin of small proteins screened in this study. 3

Table S1 C. Overview of the results of structural analysis of small proteins screened in this study. 4

Table S1 D. Classification of small protein origin screened in this study. 5

Table S2. S2D NMR chemical shift based statistical population prediction of small proteins screened in this study. 6

Table S3. Meta prediction approach of small proteins based on a combination of predictors.................... 7

Table S4. Espritz NMR predictions of small proteins screened in this study. 8

Figure S1. Sequence-based SWISS model secondary structure predictions of small proteins 9

Figure S2. Titration series of 2D $^{1}H^{15}N$-HSQC spectra of SP-23 high salt regulated protein. 10

Figure S3. Temperature series of 2D $^{1}H^{15}N$-HSQC spectra of SP-21 high zinc-binding protein. 11

Figure S4. FLYA assignment of SP-22 protein. 12

Figure S5: Progress of targeted acquisition against measurement time of SP-22 small protein. 13

Reference 14

Table S1 A. Overview of the amino acid sequences of small proteins screened in this study. The small proteins are shown according to ascending molecular weight.

ID	aa	MW, kDa	Sequence
**SP-1**	14	1.6	VSYLKRCHLAGIAR
**SP-2**	14	1.8	MANTQNISIWWWAR
**SP-3**	18	1.9	VSKKVLERGVGTTGEARL
**SP-4**	18	2.2	MLVRDLEQLLFKINLLSR
**SP-5**	23	2.6	LDSNTSHKNSVRHVLGLAQRVSF
**SP-6**	23	2.8	VPVMKNLADSMMSPMSSEARKLS
**SP-7**	27	2.9	VSRGLREGCRFSRHSASHESMPGGSHS
**SP-8**	28	3.1	MEEVNQIAGGHPTLKDGVCFGPPARLFW
**SP-9**	29	3.1	LKIAMGAGLTESRAKEAFKASKKKVAEIV
**SP-10**	31	3.7	MRPKHRRRASLFVRCKNMQECADGMAVMHIK
**SP-11**	38	4.0	MVSMRSCMCCGEPISETRHLCGVCIQNGCTSYADACGQ
**SP-12**	39	4.5	VNLMCTIAKERLQRDHWEQQAQDSVGQQEAKADKKTPTA
**SP-13**	43	4.8	MRDTAMSQRKDDHLDIVLDERTAPATVAAGRECIRFELSSDGD
**SP-14**	45	5.1	MLVMPTIDVSEHLYRQIESAADGEDLDAAMWKMVGRYQRGNTPGD
**SP-15**	46	5.1	MASDAPDGKFRSFIGRFRSQRTRLRVSACVAASRSIAVPDDDEHAE
**SP-16**	46	4.9	MSDDSNQMVTYLRQNPRMMGVLFTLTLLLSQAGSVAAGNTGIIYGP
**SP-17**	48	5.5	MKLLEMLQDLMQYFTEAFARVFGPSDDEYPAVGVQPFDGEILVNSTEE
**SP-18**	51	5.5	MNFSVVVGPRGNQHKSESGGSCRILQGSLERVGRCVASRLPLQTRRPPCVL
**SP-19**	51	5.7	MVLHNSVIDDYHPTEGYYECRSCRTRTVSASHLSECPDCGGSVRNIAVARE
**SP-20**	53	6.2	MGWIEGRDLRIGTQPLPSTSKLNFRNITSFLSFWLNPIPSTCTFIRVYIDFC
**SP-21**	59	6.5	MSESEQRHAHQCVSCGINIAGMSAATFKCPDCGQEISRCSKCRKQSNLYECPDCGFMGP
**SP-22**	60	6.7	MNKAHFEVFVDAADKYRWRLVHDNGNILADSGEGYASKQKAKQGIESVKRNAPDADVIEA
**SP-23**	61	6.9	MSSSPWTANFATEKSKCAADVQRLLEKYPQPVVYEVMSELLRQEMREQFAGAYAASQQSDD
**SP-24**	61	7.1	VTIWEYDVKEIRFSEWSKAKEDLNNLGVEGWELIKFSNEIDENGMVAAVFKRPVDYVDAAF
**SP-25**	61	7.2	MERVTLRIPKQQIEEVERMVETGEFPNRSEAIRSAVRDMLNEQVTDKRQRESTSKRGWAKV
**SP-26**	70	7.7	MAAFETTRPAPFGAISTFHFVQRMSDLLATVVAWNDARATRAALSKLSDRELDDIGLCRGDIDDICALRR
**SP-27**	78	8.1	MVYAYVMVKAAPVSDGIDQLKQDLLAVSDGIVSAHIVAGDVDFIVKVEVDSPADVKGIAGGIQSVAGIEDTQTYIAMD

Table S1 B. Overview of the origin of small proteins screened in this study. The small proteins are shown according to ascending molecular weight.

ID	collaborative ID	MW, kDa	Microorganism	Research group	University
**SP-1**	rreB	1.6	*Bradyrhizobium japonicum*	Elena Evguenieva-Hackenberg	Giessen
**SP-2**	TrpL leader	1.8	*Sinorhizobium meliloti*	Elena Evguenieva-Hackenberg	Giessen
**SP-3**	rreR	1.9	*Dinoroseobacter shibae*	Elena Evguenieva-Hackenberg	Giessen
**SP-4**	SP34_2_WW	2.2	*Methanosarcina mazei*	Ruth Schmitz	Kiel
**SP-5**	sP44	2.6	*Methanosarcina mazei*	Ruth Schmitz	Kiel
**SP-6**	SP26_1_SW	2.8	*Methanosarcina mazei*	Ruth Schmitz	Kiel
**SP-7**	na	2.9	*Sinorhizobium meliloti*	Elena Evguenieva-Hackenberg	Giessen
**SP-8**	sPP37	3.1	*Methanosarcina mazei*	Ruth Schmitz	Kiel
**SP-9**	sPP31	3.1	*Methanosarcina mazei*	Ruth Schmitz	Kiel
**SP-10**	NGR_c15640	3.7	*Sinorhizobium fredii*	Wolfgang Streit	Hamburg
**SP-11**	HVO_2983_A	4.0	*Haloferax volcanii*	Jörg Soppa	Frankfurt am Main
**SP-12**	SPR2360	4.5	*Bacillus subtilis*	Sabine Brantl	Jena
**SP-13**	NGR_a02780	4.8	*Sinorhizobium fredii*	Wolfgang Streit	Hamburg
**SP-14**	HVO_1270	4.9	*Haloferax volcanii*	Anita Marchfelder	Ulm
**SP-15**	HVO_1796	5.1	*Haloferax volcanii*	Anita Marchfelder	Ulm
**SP-16**	HVO_2354	5.1	*Haloferax volcanii*	Anita Marchfelder	Ulm
**SP-17**	Norf1 6803	5.5	*Synechocystis* sp. PCC 6803	Wolfgang R. Hess	Freiburg
**SP-18**	repX	5.5	*Sinorhizobium fredii*	Wolfgang Streit	Hamburg
**SP-19**	HVO_1533	5.7	*Haloferax volcanii*	Anita Marchfelder	Ulm
**SP-20**	sP41	6.2	*Methanosarcina mazei*	Ruth Schmitz	Kiel
**SP-21**	HVO_2753	6.5	*Haloferax volcanii*	Jörg Soppa	Frankfurt am Main
**SP-22**	HVO_2922	6.7	*Haloferax volcanii*	Anita Marchfelder	Ulm
**SP-23**	A0101	6.9	*Haloferax volcanii*	Anita Marchfelder	Ulm
**SP-24**	sP36	7.1	*Methanosarcina mazei*	Ruth Schmitz	Kiel
**SP-25**	HVO_0582	7.2	*Haloferax volcanii*	Anita Marchfelder	Ulm
**SP-26**	RSP_0557	7.7	*Rhodobacter sphaeroides*	Gabriele Klug	Giessen
**SP-27**	HVO_2212	8.1	*Haloferax volcanii*	Anita Marchfelder	Ulm

Table S1 C. Overview of the results of structural analysis of small proteins screened in this study. The small proteins are shown according to ascending molecular weight.

ID	aa	MW ,kDa	SPPS/Expression	Structural analysis
**SP-1**	14	1.6	SPPS	unstructured [1]
**SP-2**	14	1.8	SPPS	unstructured
**SP-3**	18	1.9	SPPS	unstructured [1]
**SP-4**	18	2.2	SPPS	n.a. due to hydrophobicity
**SP-5**	23	2.6	SPPS	unstructured
**SP-6**	23	2.8	SPPS	unstructured
**SP-7**	27	2.9	SPPS	unstructured
**SP-8**	28	3.1	SPPS	unstructured
**SP-9**	29	3.1	SPPS	unstructured
**SP-10**	31	3.7	SPPS	molten globule [2]
**SP-11**	38	4.0	Expression	molten globule
**SP-12**	39	4.5	Expression	molten globule
**SP-13**	43	4.8	Expression	molten globule [2]
**SP-14**	46	4.9	Expression	no expression
**SP-15**	46	5.1	Expression	no expression
**SP-16**	45	5.1	Expression	degradation
**SP-17**	48	5.5	Expression	no expression
**SP-18**	51	5.5	Expression	degradation
**SP-19**	51	5.7	Expression	structured
**SP-20**	53	6.2	Expression	degradation
**SP-21**	59	6.5	Expression	structured
**SP-22**	60	6.7	Expression	structured [3]
**SP-23**	61	6.9	Expression	molten globule
**SP-24**	61	7.1	Expression	structured
**SP-25**	61	7.2	Expression	molten globule
**SP-26**	70	7.7	Expression	degradation
**SP-27**	78	8.1	Expression	partially structured

Table S1 D. Classification of small protein origin screened in this study.

Species	Kingdom	Phylum	Class	Order	Family	Genus
Dinoroseobacter shibae	Bacteria	Proteobacteria	Alpha proteobacteria	Rhodobacterales	*Rhodobacteraceae*	*Dinoroseobacter*
Rhodobacter sphaeroides	Bacteria	Proteobacteria	Alpha proteobacteria	Rhodobacterales	*Rhodobacteraceae*	*Rhodobacter*
Sinorhizobium meliloti	Bacteria	Proteobacteria	Alpha proteobacteria	Rhizobiales	*Rhizobiaceae*	*Sinorhizobium*
Sinorhizobium fredii	Bacteria	Proteobacteria	Alpha proteobacteria	Rhizobiales	*Rhizobiaceae*	*Sinorhizobium*
Bradyrhizobium japonicum	Bacteria	Proteobacteria	Alpha proteobacteria	Rhizobiales	*Bradyrhizobiaceae*	*Bradyrhizobium*
Bacillus subtilis	Bacteria	Firmicutes	Bacilli	Bacillales	*Bacillaceae*	*Bacillus*
***Synechocystis* sp.PCC 6803**	Bacteria	Cyanobacteria		Chroococcales	*Merismopediaceae*	*Synechocystis*
Methanosarcina mazei	Archaea	Euryarchaeota	Methanomicrobia	Methanosarcinales	*Methanosarcinaceae*	*Methanosarcina*
Haloferax volcanii	Archaea	Euryarchaeota	Halobacteria	Halobacteriales	*Halobacteriaceae*	*Haloferax*

Table S2. S2D NMR chemical shift based statistical population prediction of small proteins screened in this study. [4][5] Small proteins are combined in classes with respect to experimental secondary structure screening analysis.

	ID	aa	helix	coil	extended
**folded**	**SP-19**	51	0	**1**	0
	**SP-21**	59	0	**0.95**	0.05
	**SP-22**	60	0.23	0.48	0.28
	**SP-24**	61	0.13	**0.66**	0.21
	**SP-27**	78	0.33	0.30	0.37
**molten globule**	**SP-10**	31	0.32	**0.64**	0.03
	**SP-11**	38	0.29	**0.71**	0
	**SP-12**	39	0.33	**0.67**	0
	**SP-13**	43	0	**0.86**	0.14
	**SP-23**	61	**0.71**	0.29	0
	**SP-25**	61	0.53	0.38	0.09
**unstruc-tured**	**SP-8**	28	0	**1**	0
	**SP-9**	29	**0.62**	0.38	0
**degradation**	**SP-16**	45	0.44	**0.56**	0
	**SP-18**	51	0.31	**0.61**	0.08
	**SP-26**	70	**0.56**	0.44	0
**not expressed**	**SP-14**	46	**0.59**	0.41	0
	**SP-15**	46	0.09	**0.91**	0
	**SP-17**	48	0.40	**0.60**	0

Table S3. Meta prediction approach of small proteins based on a combination of predictors (PrDOS, DisoPred2, VSL2, IUPred) [6]. Dynamic transition induced by interactions was commutated with FuzPred with a reference to metaPrDos free form. Small proteins are combined in classes with respect to experimental secondary structure screening analysis.

			Free form		Bound form	
	**ID**	**aa**	**structured**	**disordered**	**structured**	**disordered**
**folded**	**SP-19**	51	**74.5**	25.5	**92.2**	7.8
	**SP-21**	59	**78**	22	**84.7**	15.3
	**SP-22**	60	**75**	25	**93.3**	6.7
	**SP-24**	61	**90.2**	9.8	**100**	0
	**SP-27**	78	**96.2**	3.8	**100**	0
**molten globule**	**SP-10**	31	**71**	29	**96.8**	3.2
	**SP-11**	38	**81.6**	18.4	**97.4**	2.6
	**SP-12**	39	41	**59**	**64.1**	35.9
	**SP-13**	43	**62.8**	37.2	**74.4**	25.6
	**SP-23**	61	**68.9**	31.1	**75.4**	24.6
	**SP-25**	61	24.6	**75.4**	**70.5**	29.5
**unstructured**	**SP-1**	14	**64.3**	35.7	**92.9**	7.1
	**SP-2**	14	**71.4**	28.6	**92.9**	7.1
	**SP-3**	18	0	**100**	5.6	**94.4**
	**SP-5**	23	8.7	**91.3**	**69.6**	30.4
	**SP-6**	23	0	**100**	30.4	**69.6**
	**SP-7**	27	0	**100**	33.3	**66.7**
	**SP-8**	28	**64.3**	35.7	**100**	0
	**SP-9**	29	9.4	**90.6**	**93.1**	6.9
**degradation**	**SP-16**	45	**73.3**	26.7	**95.6**	4.4
	**SP-18**	51	41.2	**58.8**	**98**	2
	**SP-20**	53	**88.5**	11.5	**88.5**	11.5
	**SP-26**	70	**77.1**	22.9	**91.4**	8.6
**not expressed**	**SP-4**	18	**83.3**	16.7	**100**	0
	**SP-14**	46	**71.7**	28.3	**84.8**	15.2
	**SP-15**	46	**50**	**50**	**73.9**	26.1
	**SP-17**	48	**72.9**	27.1	**87.5**	12.5

Table S4. Espritz NMR predictions of small proteins screened in this study. [7] Dynamic transition induced by interactions was computed by the FuzPred [8] method with a reference to Espritz NMR free form. Small proteins are combined in classes with respect to experimental secondary structure screening analysis.

	ID	aa	Free form		Bound form	
			structured	disordered	structured	disordered
folded	SP-19	51	**68.6**	31.4	**92.2**	7.8
	SP-21	59	**71.2**	28.8	**84.7**	15.3
	SP-22	60	**73.3**	26.7	**100**	0
	SP-24	61	**100**	0	**100**	0
	SP-27	78	**100**	0	**100**	0
molten globule	SP-10	31	**61.3**	38.8	**93.5**	6.5
	SP-11	38	**73.7**	26.4	**100**	0
	SP-12	39	20.5	**79.4**	**41**	59
	SP-13	43	**53.5**	**46.5**	**67.4**	32.6
	SP-23	61	**67.2**	32.8	**78.7**	21.3
	SP-25	61	19.7	**80.4**	**75.4**	24.6
unstructured	SP-1	14	**78.6**	21.4	**100**	0
	SP-2	14	21.4	**78.6**	**78.6**	21.4
	SP-3	18	0	**100**	**61.1**	38.9
	SP-5	23	21.7	**78.2**	**87**	13
	SP-6	23	0	**100**	**52.2**	**47.8**
	SP-7	27	0	**100**	**63**	37
	SP-8	28	0	**100**	**57.1**	**42.9**
	SP-9	29	**79.3**	20.7	**100**	0
degradation	SP-16	45	**62.2**	37.8	**95.6**	4.4
	SP-18	51	31.4	**68.7**	**82.4**	17.6
	SP-20	53	**73.1**	26.9	**92.3**	7.7
	SP-26	70	**81.4**	18.6	**87.1**	12.9
not expressed	SP-4	18	**88.9**	11.1	**100**	0
	SP-14	46	28.3	**71.8**	**82.6**	17.4
	SP-15	46	**47.8**	**52.2**	**71.7**	28.3
	SP-17	48	**66.7**	33.3	**97.9**	2.1

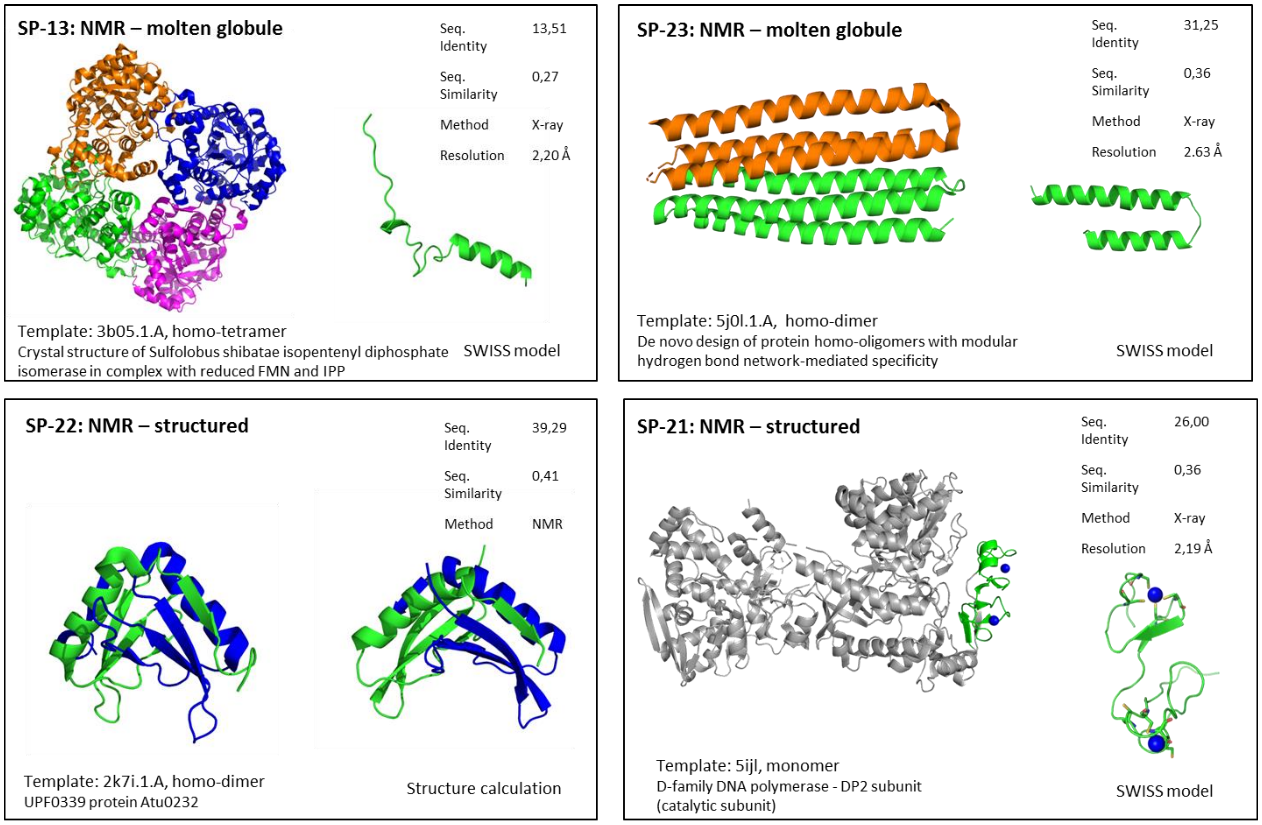

Figure S1. Sequence-based SWISS model secondary structure predictions of small proteins containing more than 30 residues. [37] NMR experiments report SP-13 and SP-23 to adopt molten globule states while SP-21 and SP-22 are folded. The homology model of SP-13 was based on the part of the homo-tetramer complex with low sequence identity (13.5%). The template used for the SWISS model structure of SP-23 protein is a homo-dimer complex (orange and green color represent the monomer subunits). Unlike the prediction, this small protein adopts a mixture of different conformational states, indicating lack of additional interaction partners. The SWISS homology model for SP-22 with the high sequence identity (39%) reports a symmetrical dimer formation (blue and green color represent the monomer subunits). The predicted geometry and fold are in good agreement with the NMR solution structure of SP-22. [3] Swiss model structure of the zinc binding protein SP-21 is defined using a catalytic part of DNA polymerase subunit as a template. It shows 26% of sequence identity. Almost all templates used for the SWISS model structures of small proteins, excluding the dimeric template for SP-22, are parts of big complexes, showing the important role of intra- and intermolecular interactions on the folding of the biological systems.

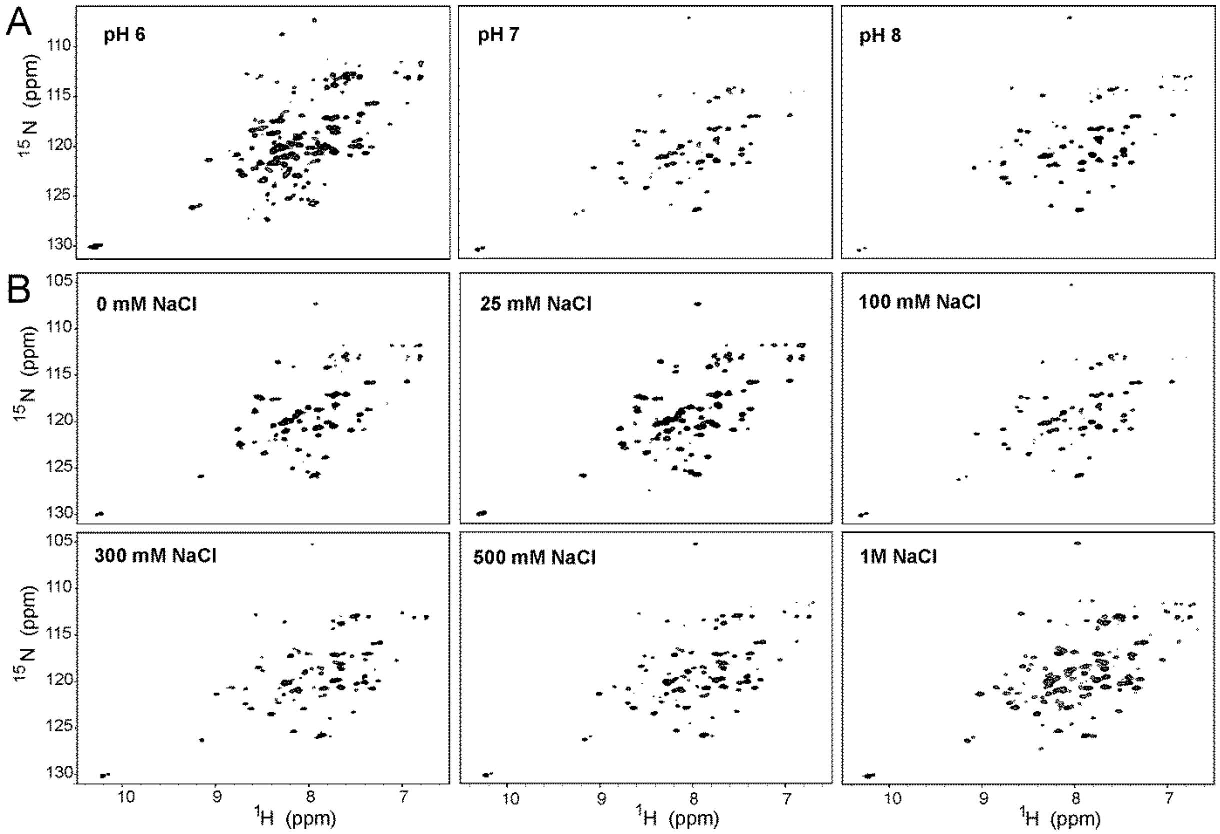

Figure S2. Titration series of 2D $^{1}H^{15}N$-HSQC spectra of SP-23 high salt regulated protein. A – pH series acquired at 100 mM NaCl. **B** – NaCl series acquired at pH 7. A and B are recorded at 700 MHz, 298 K, 25 mM phosphate buffer, pH and NaCl concentration as indicated in the figure.

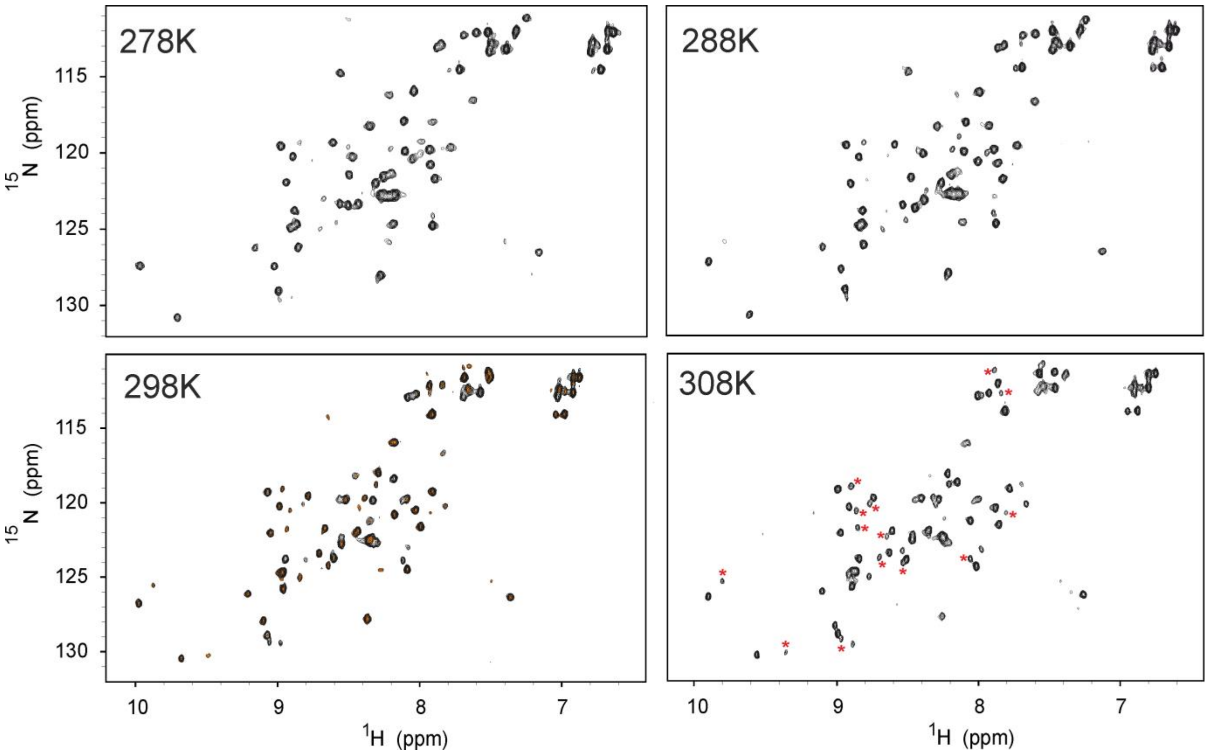

Figure S3. Temperature series of 2D $^{1}H^{15}N$-HSQC spectra of SP-21 high zinc-binding protein. 2D $^{1}H^{15}N$-HSQC temperature series from 278 to 308 K recorded at 800 MHz, 25 mM Tris pH 8, 200 mM NaCl, 3 mM DTT. The signals coming from the low populated state at 308 K are marked with red stars.

Figure S4. FLYA assignment of SP-22 protein.

A. Graphical representation of automated completed FLYA [14] assignment of SP-22 with a manually determined reference assignment.[3] Green indicate the FLYA assignment in a good agreement with the reference one, red shows atoms which do not agree with the reference, blue markers represent the FLYA assignment with no reference assignment used and black color is used for the atoms assigned in the reference but not assigned by FLYA. The assignment which is not determined to be strong is highlighted in the corresponding light colors.

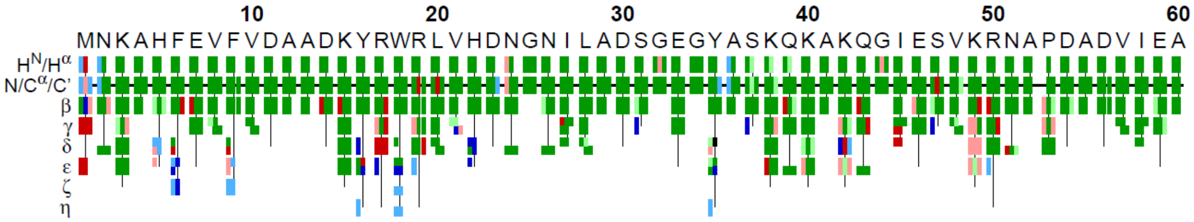

B. Automated backbone FLYA assignment of SP-22 small protein based on spectra recorded with conventional 3D NMR methods. Total measurement time is five days. Assigned 93.15%, Score 0.751.

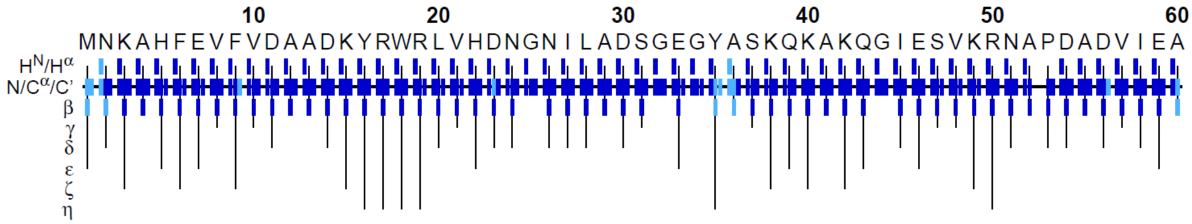

C. Automated backbone FLYA assignment of SP-22 small protein based on spectra recoded with 6% NUS amount and processed with targeted acquisition (TA) technique. Total measurement time is 4.5 hours. Assigned 90.29%, Score 0.728.

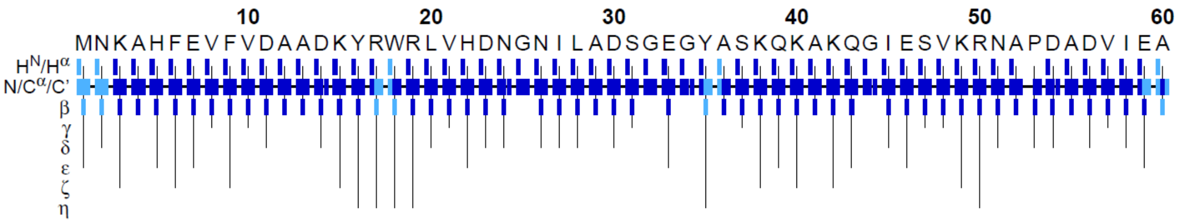

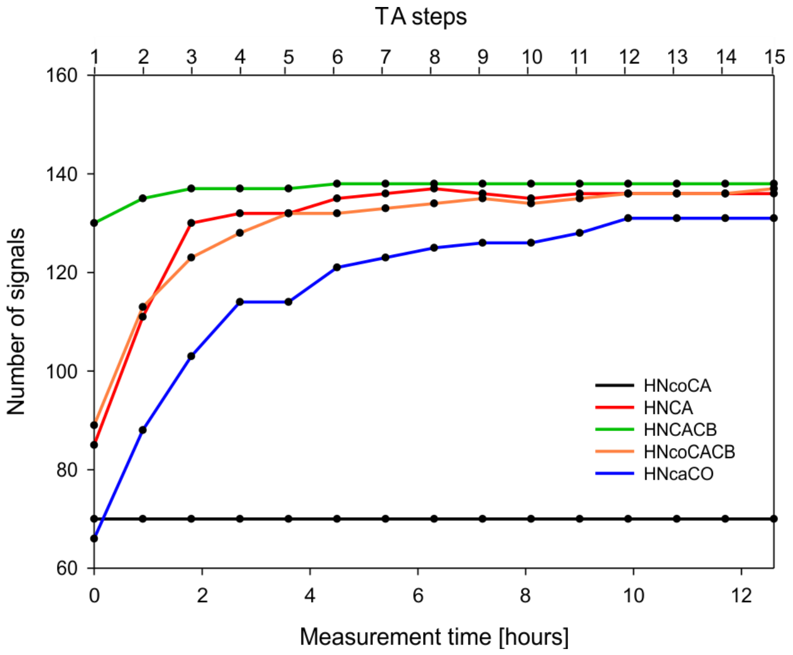

Figure S5: Progress of targeted acquisition against measurement time of SP-22 small protein. The targeted acquisition in combination with the Multi-Dimensional Decomposition signal processing technique applied on non-uniform sampled data allows an enormous reduction of the NMR measurement time. [15–17] The implemented automated FLYA assignment simplifies the evaluation of the spectra and speeds up the structural screening of small proteins. The set of spectra, recorded with NUS and targeted acquisition technique, include the following 3D heteronuclear NMR experiments: HNCO, HN(CO)CA, HNCA, HN(CO)CACB, HNCACB, HN(CA)CO. Monitoring the number of signals appearing in real-time showed that after recording of 6 TA steps corresponding to 6% NUS amount, the peak amount and therefore the quality of the recorded spectra do not change significantly anymore. This allowed to stop the spectra recording after 4.5 hours of measurement time.

Reference

[1] J. Hahn, S. Thalmann, A. Migur, R. Freiherr von Boeselager, N. Kubatova, E. Kubareva, H. Schwalbe, E. Evguenieva-Hackenberg, *RNA Biol.* **2016**, *14*.

[2] J. Grote, D. Krysciak, K. Petersen, S. Güllert, C. Schmeisser, K. U. Förstner, H. B. Krishnan, H. Schwalbe, N. Kubatova, W. R. Streit, *Front. Microbiol.* **2016**, *7*, 1858.

[3] N. Kubatova, H. R. A. Jonker, K. Saxena, C. Richter, V. Vogel, S. Schreiber, A. Marchfelder, H. Schwalbe, *ChemBioChem* **2019**, 1–9.

[4] P. Sormanni, C. Camilloni, P. Fariselli, M. Vendruscolo, *J. Mol. Biol.* **2015**, *427*, 982–996.

[5] P. Kukic, G. T. Heller, a K. Dunker, M. Bonomi, M. Fuxreiter, R. V Pappu, P. Sormanni, Z. Dosztanyi, M. M. Babu, V. N. Uversky, *Nat. Chem. Biol.* **2017**, *13*, 339–342.

[6] T. Ishida, K. Kinoshita, *Bioinformatics* **2008**, *24*, 1344–1348.

[7] I. Walsh, A. J. M. Martin, T. Di domenico, S. C. E. Tosatto, *Bioinformatics* **2012**, *28*, 503–509.

[8] M. Miskei, C. Antal, M. Fuxreiter, *Nucleic Acids Res.* **2017**, *45*, D228–D235.

[9] N. Guex, M. C. Peitsch, T. Schwede, *Electrophoresis* **2009**, *30*, 162–173.

[10] P. Benkert, M. Biasini, T. Schwede, *Bioinformatics* **2011**, *27*, 343–350.

[11] M. Bertoni, F. Kiefer, M. Biasini, L. Bordoli, T. Schwede, *Sci. Rep.* **2017**, *7*, 1–15.

[12] S. Bienert, A. Waterhouse, T. a. P. De Beer, G. Tauriello, G. Studer, L. Bordoli, T. Schwede, *Nucleic Acids Res.* **2017**, *45*, D313–D319.

[13] A. Waterhouse, M. Bertoni, S. Bienert, G. Studer, G. Tauriello, R. Gumienny, F. T. Heer, T. a. P. De Beer, C. Rempfer, L. Bordoli, *Nucleic Acids Res.* **2018**, *46*, W296–W303.

[14] E. Schmidt, P. Güntert, *J. Am. Chem. Soc.* **2012**, *134*, 12817–12829.

[15] V. A. Jaravine, A. V. Zhuravleva, P. Permi, I. Ibraghimov, V. Y. Orekhov, *J. Am. Chem. Soc.* **2008**, *130*, 3927–3936.

[16] V. Y. Orekhov, V. A. Jaravine, *Prog. Nucl. Magn. Reson. Spectrosc.* **2011**, *59*, 271–292.

[17] L. Isaksson, M. Mayzel, M. Saline, A. Pedersen, J. Rosenlöw, B. Brutscher, B. G. Karlsson, V. Y. Orekhov, *PLoS One* **2013**, *8*, 1–8.
